# Gastrointestinal Tract Findings in Patients with Primary Immunodeficiency: A Single-Center 6-Year Experience*

**DOI:** 10.5152/tjg.2025.25100

**Published:** 2025-08-25

**Authors:** Merve Erkoç, Çiğdem Erhan, Leyla Çevirme, Hakan Basır, Susamber Dik, Reyhan Sevil Cansunar, Güzin Özden

**Affiliations:** 1 Division of Immunology and Allergy Diseases, Adana City Training and Research Hospital, Adana, Türkiye; 2 Department of Internal Medicine, Adana City Training and Research Hospital, Adana, Türkiye

**Keywords:** Chronic diarrhea, common variable immunodeficiency, gastric carcinoma, gastrointestinal tract, primary immunodeficiency, switched memory B cell

## Abstract

**Background/Aims:**

The aim was to determine the findings of the gastrointestinal system, which is the second most frequently affected system in the primary immunodeficiency (PID) patient population, and the frequency of these findings.

**Materials and Methods:**

Fifty patients with PID were included in this study, and the characteristics of the patients, upper gastrointestinal endoscopy, colonoscopy, and biopsy (endoscopic and colonoscopic) results were evaluated.

**Results:**

The median age of patients included in the study was 31 years (range 18-72 years) and 64% were male. Seventy-two percent of the patients had common variable immunodeficiency (CVID) and 68% were diagnosed in adulthood. Chronic diarrhea was present in 48% of the patients, and body mass index was lower in this group. Switched memory B cells were lower in chronic diarrhea (*P* = .003). Twenty-nine patients underwent upper gastrointestinal endoscopy, and the most common macroscopic findings were gastropathy (79.3%), duodenopathy (37.9%), and esophagitis (27.6%). Of the 23 patients who underwent colonoscopy, 14 had at least 1 macroscopic finding other than internal hemorrhoids and only 1 patient had no macroscopic findings. One patient had mucosa-associated lymphoid tissue lymphoma (MALToma) on gastric biopsy, while 1 patient had poorly differentiated adenocarcinoma on antrum biopsy.

**Conclusion:**

In conclusion, chronic diarrhea is more common in PID than in the general population, and switched memory B cells are lower in PID patients with chronic diarrhea. Most importantly, a collaboration between immunologists, gastroenterologists, and pathologists is required when evaluating the gastrointestinal tract in PID.

**Main Points**

- Gastrointestinal tract manifestations, including chronic diarrhea, are common in patients with primary immunodeficiency (PID).
- Switched memory B cells were statistically significantly lower in PID patients with chronic diarrhea.
- Further studies are needed on gastrointestinal tract findings in patients with PID in collaboration with immunologists, gastroenterologists, and pathologists.

## Introduction

Primary immunodeficiency (PID) diseases are now called Inborn Errors of Immunity (IEI) and are caused by defects in various areas of the immune system.^1^ The International Union of Immunology Societies (IUIS) has classified IEI according to clinical and laboratory features in its 2022 update. There are 485 IEIs in this classification, which describes various underlying phenotypes such as infection, malignancy, allergy, autoimmunity, and autoinflammation.^2^ Common variable immunodeficiency (CVID) is the most common symptomatic PID in adults and is associated with a defect in antibody production resulting from the failure of B cell differentiation. In CVID, a decrease in switched memory B cells, which are a subgroup of B lymphocytes, is observed.^3^^,^^4^Although the most common manifestation of IEI is respiratory symptoms, the gastrointestinal (GI) system is the second most common system in which complications occur. Studies report that the prevalence of GI symptoms in patients with IEI varies between 5% and 50%.^5^ Infections and non-infectious diarrhea in the GI system are common, but malignancies, inflammatory findings, and autoimmune-related diseases have also been described. Even GI involvement may be a symptom of the disease in these patients.^6^ Studies on GI manifestations in patients with PID in different populations are included in the literature.^5^^,^^7^^-^^10^ The aim was to determine the GI tract findings and the prevalence of findings in PID patients in our population.

## Materials and Methods

### Study Population and Design

**Figure 1.**
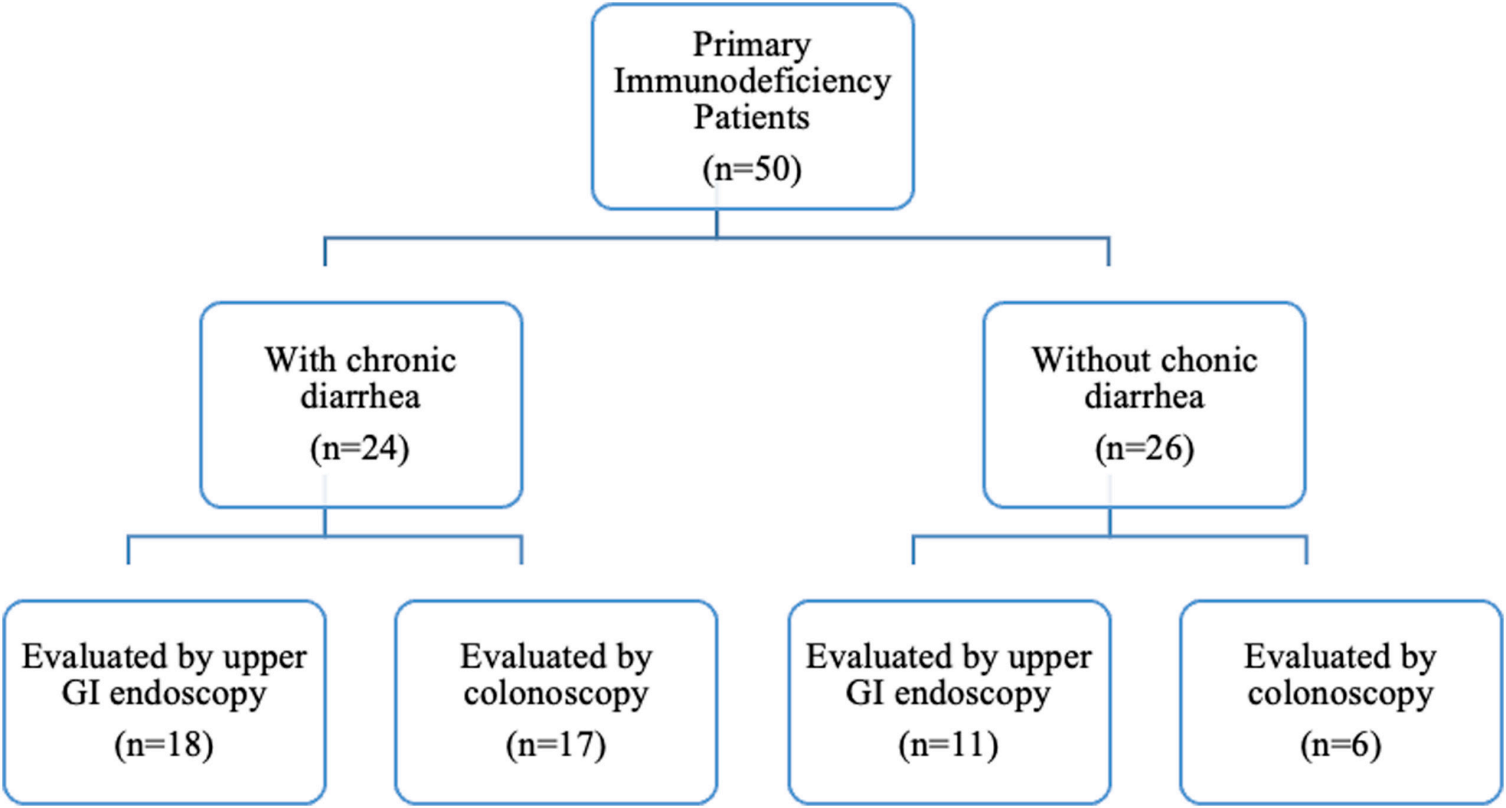
The study flow diagram.

Fifty patients diagnosed with PID using the European Society for Immunodeficiency Diseases criteria^4^ and followed up in the immunology and allergy outpatient clinic of Adana City Training and Research Hospital between December 2017 and December 2023 were included in this study. In addition, the patients were classified according to the International Union of Immunological Societies(IUIS) classification.^2^ The files of the patients included in the study were reviewed. Demographic characteristics, clinical information, and laboratory values of the patients were recorded. Patients were evaluated for organomegaly by abdominal ultrasonography. Chronic diarrhea was defined as decreased stool consistency or increased stool frequency lasting longer than 4 weeks.^11^ Twenty-nine patients underwent upper GI endoscopy and 23 patients underwent colonoscopy. The flow of the study is shown in Figure 1. Biopsy findings obtained during upper GI endoscopy and colonoscopy were recorded. Approval for the study was obtained from the Clinical Research Ethics Committee of Adana City Training and Research Hospital (Decision No.: 3110; Date: January 18, 2024). Since patient files were reviewed retrospectively, informed consent was not obtained.

### Laboratory Evaluation

Serum immunoglobulin (Ig) levels were determined using the nephelometric method. Low immunoglobulin A and low IgM levels were defined as <2 SDs from normal levels for age on at least 2 measurements of IgA and IgM.^4^ Lymphocyte subsets, including cluster of differentiation (CD)3^+^ T cells (including separate counting of CD4^+^ T helper cells and CD8^+^ T cytotoxic cells), CD19^+^ B cells, CD16^+^56^+^ natural killer (NK) cells, and CD4^+^/CD8^+^ ratio, were enumerated by flow cytometry. Switched memory B cells are identified by the presence of the CD27 memory marker and also by the loss of IgD and IgM expression because they have undergone isotype switching. Additionally, CD21low B cells, a heterogeneous B cell subset expanded in a wide variety of autoimmune diseases and splenomegaly, were assessed by flow cytometry.^12^

### Statistical Analysis

The data obtained were evaluated statistically using IBM SPSS (Statistical Package for Social Sciences) version 27 (IBM SPSS Corp.; Armonk, NY, USA), and *P* < .05 was considered statistically significant. In descriptive statistics, categorical variables are shown as frequency and percentage. Metric variables that do not show a normal distribution are given as median (minimum-maximum), and metric variables that show a normal distribution are given as mean ± SD. The chi-square test was used to compare independent groups for categorical variables. The Student’s *t*-test was used to compare normally distributed metric variables, while the Mann–Whitney *U* test was used to compare non-normally distributed metric variables.

## Results

### Characteristics of Patients

The median age of the 50 patients included in the study was 31 years (range 18-72 years) and 64% were male. Sixty-eight percent of patients were diagnosed in adulthood, and the diagnostic delay was 6.5 years (range 0-51 years). There was a history of consanguineous marriage between parents in 29.5% of the patients, and the family history of immunodeficiency was 28.3%. When comparing those with and without chronic diarrhea, the proportion of patients with low IgA or IgM was higher in those with chronic diarrhea, but this was not statistically significant. Splenomegaly was more common in the chronic diarrhea group (*P* = .049). When lymphocyte subgroups were examined, switched memory B cells were lower in the chronic diarrhea group, and this was statistically significant (*P* = .003), and CD21low B cells were higher, but this was not statistically significant (*P* = .289). Demographic information, clinical characteristics, and laboratory values of the patients are shown in Table 1. All patients in the study were receiving immunoglobulin replacement therapy. The majority of patients had predominantly antibody deficiency (PAD), and 72% had CVID. The IUIS phenotypic classification of the patients included in the study is given in Table 2. Fifty percent of CVID patients had chronic diarrhea, 5.6% had gluten enteropathy, 2.8% had inflammatory bowel disease (IBD), and 5.6% had GI-related malignancies.

### Upper Gastrointestinal Endoscopy Findings

Upper GI endoscopy was performed in 29 patients, and all patients had at least 1 macroscopic finding. Upper GI endoscopy was performed in 75% of patients with chronic diarrhea. Gastropathy (79.3%) was the most common macroscopic finding observed in upper GI endoscopy, followed by duodenopathy (37.9%) and esophagitis (27.6%). Upper GI endoscopic findings of the patients are given in Table 3.

### Colonoscopy Findings

Seventeen of the patients who underwent colonoscopy had chronic diarrhea. Fourteen of the 23 patients who underwent colonoscopy had at least 1 macroscopic finding other than internal hemorrhoids. Only 1 patient had no macroscopic findings. Polyps were detected in 2 patients during colonoscopy; one of them was in the rectum, and the other was in the colon. The colonoscopy findings of the patients are shown in Table 3.

### Histopathological Findings

Gastric biopsy results were available for 16 patients, and 6 patients had *Helicobacter pylori* (*H. pylori*) positivity in their biopsy results. In the gastric biopsies, atrophy was detected in 4 patients, and intestinal metaplasia was present in 4 patients. In 1 patient, mucosa-associated lymphoid tissue lymphoma (MALToma) accompanying *Helicobacter pylori* gastropathy was observed in the gastric biopsy, while in 1 patient, poorly differentiated adenocarcinoma was present in the antrum biopsy and was evaluated as a signet ring cell. Of the polyps, the 1 in the rectum was an adenomatous polyp with tubular adenoma morphology, while the 1 in the colon was a tubulovillous adenoma with high-grade dysplasia in the glandular epithelium.

## Discussion

In this study, upper GI endoscopy and colonoscopy findings were shared in 50 patients followed up with a diagnosis of PID, almost half of whom had chronic diarrhea. When patients with chronic diarrhea were compared with patients without chronic diarrhea, the age of diagnosis was later and the proportion of patients diagnosed in adulthood was higher. The frequency of GI findings in PIDs is observed to be quite variable.^5^^,^^7^^,^^13^^-^^15^ The reason for this variability can be considered as the heterogeneity of immunodeficiency phenotypes of the cohorts included in the studies and the evaluation of different GI findings. Chronic diarrhea, 1 of the GI findings, affects up to 5% of the adult population and is considered a common problem.^11^ It is also frequently seen in PIDs.^7^^,^^16^ In CVID, 20% to 60% of patients experience chronic or intermittent diarrhea, depending on the cohort.^17^ In this study, 48% of the patients had a history of chronic diarrhea, while this rate was 50% in CVID. Although the frequency of chronic diarrhea varies in different PID populations and phenotypes, the most important point is that the frequency of chronic diarrhea appears to be increased in patients with PID compared to the general population. Furthermore, chronic diarrhea is the most common GI symptom associated with PADs, especially non-infectious diarrhea, leading to diagnostic delays and poor prognosis.^18^ And regardless of the underlying pathogenesis, chronic diarrhea carries a poor prognosis, resulting in malabsorption, protein-energy malnutrition, electrolyte imbalances, and failure of immunoglobulin replacement therapy.^17^ As an expected result, the body mass index of patients with chronic diarrhea was lower, although not statistically significant. It was also observed that the age of diagnosis was later in patients with chronic diarrhea. When the effect of chronic diarrhea on prognosis is considered, it is thought that increasing awareness in physicians who care for this patient population can contribute to the prognosis. Furthermore, in chronic diarrhea, inflammatory and infectious conditions often overlap, further complicating diagnosis and treatment.^17^ This complexity, together with the lack of data in this study, prevented detailed information on infectious diarrhea. Previous studies have shown that low B cells (CD19^+^) and low memory B cells in CVID are associated with inflammatory GI manifestations.^15^^,^^19^ Additionally, 1 study found that low B cells and low switched memory B cells were associated with autoimmune/inflammatory GI manifestations.^20^ It has also been shown that memory B cells are lower in patients with PAD and chronic diarrhea.^21^ In this study, both switched memory B cells and B cells (CD19^+^) were found to be low in patients with chronic diarrhea in the PID group, which also covers CVID, and only the switched memory B cell low was statistically significant. Switched memory B cell reduction was observed more in the CVID group, and no significant difference was observed between the chronic diarrhea and non-diarrhea groups in the CVID patient population. In this case, it is believed that chronic diarrhea in PIDs, independent of CVID, is associated with B lymphocytes (CD19^+^) and especially switched memory B lymphocytes. All patients who underwent endoscopy had stomach macroscopic findings. Gastropathy was the most common (79.3%) among gastric macroscopic findings, and gastropathy was accompanied by ulcers in 4 patients. Chronic gastritis also occurs in CVID, and the most common complication is thought to be atrophic gastritis, affecting approximately 30%.^9^ In this study, atrophic gastritis was observed at a lower rate (11.5%) than expected at endoscopy in CVID, but the addition of histopathological evaluation to this evaluation will increase the frequency. As in previous studies, intestinal metaplasia was also detected histopathologically in CVID patients undergoing endoscopy.^22^^,^^23^ The observation of areas of atrophic gastritis, intestinal metaplasia, or dysplasia in gastric histology obtained during upper endoscopy before the onset of cancer,^22^ suggests that these patients, who are already at risk for GI malignancy, should be regularly screened for GI tracts at certain intervals.

In Italy, 56% of patients with PAD had at least a visible change on colonoscopy, the most common being colon polyps (53.2%), followed by hemorrhoids (16.5%) and mucosal edema (11.4%).^24^ In this study, 95.7% of the patients who underwent colonoscopyhad at least 1 macroscopic finding, and hemorrhoids (65.2%) were the most common, followed by colitis (26.1%) and terminal ileitis (26.1%), respectively. The majority of patients in this study had PAD, and this may be due to the different populations. In this study, the polyp rate was found to be lower compared to previous studies conducted with both PID^24^ and CVID.^15^^,^^23^ Histopathologically, as in this study, colon tubular adenoma and tubulovillous adenoma are also seen in PIDs, but their frequencies vary.^5^^,^^23^^,^^24^ The worldwide prevalence of hemorrhoids varies between 4% and 55%, and there are many factors that affect its occurrence, including eating habits, lack of physical exercise, defecation behavior, and genetic factors.^25^ In this study, the incidence of hemorrhoids was found to be 65.2%, which is higher than both PIDs^24^ and the worldwide prevalence.^25^ The various results observed at colonoscopy may be due to many factors, including regional differences in dietary habits and genetic factors.

There is an increased incidence of IBD in patients with CVID and even an increased incidence of PID (including CVID) in patients with early-onset IBD.^26^ However, the frequency of IBD in patients with CVID varies across studies,^15^^,^^20^^,^^23^^,^^27^^,^^28^ and early-onset IBD was also observed in one of the CVID patients. Gluten enteropathy also occurs in CVID, and its incidence varies considerably across various CVID cohorts, including this study.^20^^,^^28^^,^^29^ It is difficult to distinguish CVID-associated enterocolitis from IBD and CVID-associated enteropathy from gluten enteropathy because of their clinical and histopathological mimics.^26^ This may have accounted for the difference in diagnosis frequency between populations. It is crucial for gastroenterologists, pathologists, and immunologists to collaborate closely in the diagnosis of these diseases. Malignancies are the second most common cause of death in PID patients after infection, and GI system malignancies are seen in PID patients.^5^^,^^30^Although no significant increase in other malignancies was observed in CVID, an increased incidence of non-Hodgkin lymphoma and gastric cancer was reported.^22^ However, the mechanisms underlying the increased incidence of gastric cancer remain unclear.^31^ However, *H. pylori* has been associated with gastritis, gastric dysplasia, and gastric cancer in this patient group, and it is thought that the frequency of *H. pylori* infection in patients with CVID is similar to the general population.^6^ In this study, *H*. *pylori* positivity was detected in 6 of 16 patients, which was more frequent than in previous studies,^32^^, 28^ and MALToma accompanying *H*.* pylori* gastropathy was observed in the stomach biopsy of one of these patients. Gastric cancer was also observed in this study, as reported in the literature.^15^^,^^22^^,^^28^ Although it can be seen in PIDs, it should be noted that the tendency to GI system malignancies increases especially in CVIDs and that early diagnosis can save the lives of patients. In addition, *H. pylori* eradication is very important in this patient population.

This study is one of the few studies conducted in the country with GI tract findings in PID in adults, and the inclusion of flow cytometry strengthens the study. In particular, the relationship between chronic diarrhea and switched memory B cells in PID as a result of this study will contribute to the literature. With the increase in studies on this subject, it is believed that switched memory B cells can provide information about the diagnosis, course, and response to treatment of gastrointestinal diseases.

The first limitation of this study is that, although it is a good number of patients with PID from a single center, a limited number of patients were included in the study. The second limitation is that since patient information was evaluated retrospectively from the records, all GI findings could not be clearly obtained, thus causing a lack of data. Lack of data has also resulted in a lack of information on whether chronic diarrhea is infectious. The third limitation is that all biopsies of patients with macroscopic findings on endoscopy and colonoscopy could not be accessed. In conclusion, PID patients with chronic diarrhea have a low number of switched memory B cells, and GI symptoms, including chronic diarrhea, are more common in PID patients than in the general population. However, both populations and studies are heterogeneous, and study results are highly variable. It is clear that further studies, including collaborative approaches between immunologists, gastroenterologists, and even pathologists, are needed to better understand this issue.

## Figures and Tables

**Table 1.** Demographic Information, Clinical Characteristics, and Laboratory Values of the Patients

Yes (n = 24)		No (n = 26)
Characteristics	Overall (n = 50)	Chronic Diarrhea		*P*
		Age, years^*^	31.0 (18.0-72.0)		35.5 (19.0-64.0)	28.0 (18.0-72.0)	.690
Gender, n (%) Female Male	18.0 (36.0) 32.0 (64.0)	7.0 (29.2) 17.0 (70.8)	11.0 (42.3) 15.0 (57.7)	.333
BMI^*^	22.3 (12.0-35.2)	21.4 (14.9-31.2)	23.8 (12.0-35.2)	.080
Age of symptom onset, years^*^	12.0 (0.0-53.0)	8.5 (0.0-53.0)	12.0 (0.0-48.0)	.738
Age at diagnosis, years^*^	25.0 (0.0-61.0)	32.0 (0.0-60.0)	20.0 (0.0-61.0)	.330
Delay in diagnosis, years^*^	6.5 (0.0-51.0)	7.0 (0.0-37.0)	6.0 (0.0-51.0)	.140
Time to diagnosis, n (%) Childhood Adulthood	16 (32.0) 34 (68.0)	6 (25.0) 18 (75.0)	10 (38.5) 16 (61.5)	.308
Consanguineous marriage (n = 44), n (%)	13 (29.5)	6 (25.0)	7 (26.9)	.176
Family history of immunodeficiency (n = 46), n (%)	13 (28.3)	5 (25.0)	8 (30.8)	.087
Diagnostic presentation, n (%) Infection Chronic diarrhea Autoimmunity Splenomegaly Others	39 (78.0) 3 (6.0) 3 (6.0) 2 (4.0) 3 (6.0)	19 (79.2) 3 (12.5) 0 (0.0) 1 (4.2) 1 (4.2)	20 (76.9) 0 (0.0) 3 (11.5) 1 (3.8) 2 (7.6)	.179
Gastrointestinal findings, n (%) Chronic diarrhea Gluten enteropathy Inflammatory bowel disease Malignancy	24 (48.0) 4 (8.0) 1 (2.0) 2 (4.0)	24 (100.0) 4 (16.7) 1 (4.2) 1 (4.2)	0 (0.0) 0 (0.0) 0 (0.0) 1 (3.8)	.233
Common variable immunodeficiency, n (%)	36 (72.0)	18 (75.0)	18 (69.2)	.650
Splenomegaly, n (%)	24 (48.0)	15 (62.5)	9 (34.6)	**.049**
Hepatomegaly, n (%)	14 (28.0)	6 (25.0)	8 (30.8)	.650
Low IgA, n (%)	43 (86.0)	23 (95.8)	20 (76.9)	.054
Low IgM, n (%)	34 (68.0)	19 (79.2)	15 (57.7)	.104
Lymphocyte subsets in flow cytometry CD3^+^ T cell^*^ CD3^+^CD4^+^ T cell^ **^ CD3^+^CD8^+^ T cell^ **^ CD19^+^ B cell^*^ CD16^+^56^+^ NK cell^*^ CD4/CD8 ratio^*^ Class switched memory B cell^*^ CD21low B cell^*^	83.9 (24.0-96.5) 29.8 ± 12.3 47.6 ± 16.6 6.0 (0.0-46.1) 6.5 (0.4-38.5) 0.7 (0.0-2.7) 0.3 (0.0-56.9) 7.5 (0.0-73.9)	85.2 (24.0-96.5) 28.5 ± 11.2 49.3 ± 15.4 4.0 (0.0-11.6) 6.1 (2.3-38.5) 0.6 (0.2-2.7) 0.0 (0.0-25.0) 8.8 (0.0-73.9)	83.0 (33.6-95.5) 30.9 ± 13.4 46.1 ± 17.8 7.8 (0.0-46.1) 7.0 (0.4-35.2) 0.7 (0.0-2.5) 1.1 (0.0-56.9) 7.5 (0.0-45.0)	.514 .862 .266 .118 .182 .907 **.003** .289

BMI, body masss index; CD, cluster of differentiation; Ig, immunoglobulin; NK, natural killer; IgA, Immunoglobulin A; IgM, Immunoglobulin M.

*Median (min-max).

**Mean ± SD.

*P* < .05 was considered statistically significant.

**Table 2.** International Union of Immunology Societies Phenotypic Classification of Patients

Immunodeficiency affecting cellular and humoral immunity, n (%) Severe combined immunodeficiency Hyper IgM deficiency	1 (2.0) 1 (2.0)
Combined immunodeficiency with associated or syndromic features, n (%) Ataxia telangiectasia Hyper IgE syndrome	1 (2.0) 1 (2.0)
Predominantly antibody deficiency, n (%) X-linked agammaglobulinemia Common variable immunodeficiency	5 (10.0) 36 (72.0)
Diseases of immune dysregulation, n (%) LRBA deficiency CTLA-4 deficiency	2 (4.0) 2 (4.0)
Congenital defects of phagocyte number, function, or both, n (%) Chronic granulomatous disease	1 (2.0)

CTLA, cytotoxic T-lymphocyte-associated protein; Ig, immunoglobulin; LRBA, lipopolysaccharide-responsive beige-like anchor.

**Table 3.** Upper Gastrointestinal Endoscopy and Colonoscopy Findings

Upper GI Endoscopy Findings	Total (n = 29), n (%)	CVID (n = 26), n (%)
Esophagus Esophagitis Ulcer	8 (27.6) 1 (3.4)	8 (30.8) 1 (3.8)
Stomach Gastropathy Gastropathy-atrophic Gastropathy-erosive Gastric ulcer	23 (79.3) 4 (13.8) 2 (6.9) 4 (13.8)	21 (80.8) 3 (11.5) 2 (7.7) 4 (15.4)
Duodenum Duodenopathy	11 (37.9)	9 (34.6)
**Colonoscopy Findings**	**Total (n = 23), ** **n (%)**	**CVID (n = 21), ** **n (%)**
Ileum Terminal ileitis İleitis and ulcer	6 (26.1) 2 (8.7)	6 (28.6) 2 (9.5)
Colon Colitis Polyp Angiodysplasia	6 (26.1) 2 (8.7) 1 (4.3)	6 (28.6) 2 (9.5) 1 (4.8)
Anal canal Hemorrhoids Anal prolapse	15 (65.2) 1 (4.3)	13 (61.9) 1 (4.8)

CVID, common variable immunodeficiency; GI, gastrointestinal.

## Data Availability

The data that support the findings of this study are available on request from the corresponding author.
